# Editorial: Micronutrients: The Borderline Between Their Beneficial Role and Toxicity in Plants

**DOI:** 10.3389/fpls.2022.840624

**Published:** 2022-02-11

**Authors:** Antonios Chrysargyris, Monica Höfte, Nikos Tzortzakis, Spyridon A. Petropoulos, Francesco Di Gioia

**Affiliations:** ^1^Department of Agricultural Sciences, Biotechnology and Food Science, Cyprus University of Technology, Limassol, Cyprus; ^2^Department of Plants and Crops, Ghent University, Ghent, Belgium; ^3^Department of Agriculture, Crop Production and Rural Environment, University of Thessaly, Magnissia, Greece; ^4^Department of Plant Science, The Pennsylvania State University, University Park, PA, United States

**Keywords:** mineral nutrition, plant metabolism, human health, toxicity, deficiency

## Micronutrients: Introduction

Plant nutrition management in commercial crops through the application of macro and micronutrients is essential not only for achieving high yields but also for fulfilling market requirements for high quality end-products (Sarwar et al., 2010). Common nutrition practices focus on the application of macronutrients through synthetic fertilizers without considering micronutrients (Baldantoni et al., 2019). In addition, it is not uncommon the irrational use of excessive fertilizer rates which may result in soil and/or ground water contamination and phytotoxicity (Ayoub, 1999). Recent research focusing on crop micronutrient management revealed several mechanisms involved in micronutrients' uptake and translocation in plants, and their role in plant physiological processes (Thapa et al., 2021). Moreover, several reports suggest specific strategies that could help toward the optimization of micronutrients application management in modern crop production (Shukla et al., 2021).

Apart from their role in plant growth and development, micronutrients are also essential in plant tolerance against stressors and in plant innate immunity, by being involved in metabolic processes that control plant response and perception to stressors (Jan et al., 2022). However, despite the well-proven beneficial role of micronutrients, excessive applications of such nutrients may lead to plant toxicity, soil contamination and environmental problems, and detrimental health effects (Rehman et al., 2019). Moreover, considering that plant-derived micronutrients are essential for human health, another important aspect in crop nutrition is the biofortification of end-products with micronutrients that could help fighting against mineral deficiencies that plague a great part of world population (Di Gioia et al., 2019).

The present Research Topic gathers several studies that focus on the beneficial role of micronutrients in plant nutrition, while aiming at the same time to set the borderlines between excessive use and deficiencies in plant development.

## Micronutrients: The Borderline Between Beneficial Roles and Toxicity in Plants

The review article of Bai et al. provides insights into the function, transport, and signal transduction of mineral nutrients associated with Rosaceae fruit quality, and postharvest storage at physiological and molecular levels. The role of nitrogen (N), phosphorus (P) and iron (Fe) was pointed, affecting fruit quality, interaction with other minerals and increasing oxidative stress response and antioxidant enzymes activities. This knowledge will contribute to provide theoretical basis to improve fertilizer use efficiency and the sustainable development of the fruit industry. Crop micronutrients uptake is influenced by macronutrients levels. In this context, Chen et al. studied the effect of P in hydroponically grown pear seedlings, observing that higher P rates decreased *zinc (Zn), copper (Cu)* and *manganese (Mn)* and enhanced *boron (B)* and to some extent *Fe* content. *Chlorine (Cl)* is another micronutrient essential for plant growth. Liu et al. investigated the importance of Cl-containing fertilizers on citrus production and reported increased yield, vitamin C, and improved flavor and juice yield of citrus via enhanced N and K content. Moreover, the application of Cl-containing fertilizer may limit the risk of excessive sulfur (S) in citrus production. *Zinc* biofortification and the importance of Zn in human health is reported by Ali et al., who evaluated the effects of a bioactive nutrient fortified fertilizer containing Zn on 15 wheat varieties. The study revealed that beneficial microbes coated on urea were an effective biofortification means for Zn, resulting in increased Zn uptake and wheat yield. Xue et al. reported that Zn accumulation in maize improved with increasing N rates. The nitrogen form also affected the accumulation of Zn, with NH${}_{4}^{+}$ nutrition being effective in enhancing tolerance of maize seedlings to Zn-deficiency stress.

Another important micronutrient is Fe whose deficiency can cause chlorosis in many crops. Li, Cao et al. studied Fe deficiency in *Areca catechu* (L.) seedlings and reported chloroplast degeneration and reduced chlorophyll synthesis in chlorotic leaves. Iron-deficient plants showed a down-regulation of nitrate reductase and glutamate synthase gene expression but accumulated more organic acids and flavonoids. In the case of Fe excess, peroxidase-related genes were upregulated as a defense strategy against Fe toxicity.

*Manganese* is an essential micronutrient for plant growth as it is involved in the structure of photosynthetic proteins and enzymes. However, Mn deficiency might be observed in dry, calcareous and sandy soils, resulting in crop yield reduction. Ijaz et al. studied Mn solubilization and the ability of *Bacillus* spp. strains to solubilize Mn. The Mn-solubilizing bacterial strains were isolated from the maize rhizosphere and could be used as potential bioinoculants to promote plant growth under Mn deficiency. On the other hand, Mn in excess can be toxic to plants as reported by Li, Dong et al., who suggested that metal tolerance proteins (MTPs) may play a critical role in Mn tolerance in plants, including soybean (*Glycine max*). In the same study, it was demonstrated that GmMTP8.1, an endoplasmic reticulum-localized Mn transporter, contributes to confer Mn tolerance by stimulating export of Mn out of leaf cells and increasing sequestration of Mn into intracellular compartments.

*Boron* is another key micronutrient required for plant growth and development, but causing severe symptoms of phytotoxicity when applied in excess. Khan et al. investigated different B levels in 19 *Aegilops* accessions and one bread wheat cultivar. The impact of B toxicity stress affected growth parameters, with more pronounced effects on shoots rather than roots. In this study, it was also proposed that some of the studied *Aegilops* accessions could potentially be used for developing introgression lines or as pre-breeding material to genetically improve B toxicity-tolerance traits. In another study, Rékási et al. reported that in tomato, green bean, potato, and cabbage irrigated with water containing 0.1 or 0.5 mg/L of B and grown in different soil types, the accumulation of B in plant tissue was influenced by plant species and soil type. Moreover, irrigation with 0.1 mg/L B accelerated tomato fruit ripening and doubled chlorophyll content while 0.5 mg/L B negatively affected green beans nutritional value. Additionally, Pereira et al. reported that when B is absorbed by the roots, it is preferably distributed to developing tissues, such as meristems and reproductive organs. This highlights the potential role of B in mediating plant development programs, by promoting the transition from the vegetative to reproductive phase, as well as enabling land plants to complete their life cycle. Indeed, understanding the mechanisms behind the accepted (and potential) functions of B may help to elucidate how and to what extent B is an important element for plants.

Agronomic biofortification is a new approach used to enhance mineral content in plant tissue and as such may prevent nutritional deficiencies and chronic diseases in humans. Examining the biofortification of several herbs with *selenium (Se)*, Newman et al. found that biofortification not only increased Se levels, but also enhanced total phenols and antioxidant capacity, turning crops tested into functional food. Rakoczy-Lelek et al. also reported on the effectiveness of foliar biofortification of carrots with Se and *iodine (I)* and found that levels of Se and I translocated from leaves to the storage roots were within ranges considered safe for consumption. However, no synergistic or antagonistic interaction between Se and I was observed in terms of biofortification effectiveness in roots, suggesting the possibility to biofortify carrots with multiple minerals. Additional work on I uptake, translocation and metabolism in lettuce was performed by Smoleń et al. by testing different sources of I whether or not combined with vanadium (V) fertilization. The study revealed that several genes (i.e., *per64-like, samdmt, msams5*, and *cipk6*) played a functional role. It was proposed that the protein encoded by *cipk6* may function as a triiodothyronine (T3) or thyroxine (T4) receptor, mainly in lettuce roots. Additionally, the *per64-like*, rather than the *per12-like* gene, may encode a V-dependent haloperoxidase (vHPO), an enzyme that participates in I uptake. New evidence for the nutritional role of I in plants is reported by Kiferle et al. I specifically regulates the expression of genes involved in the plant defense response, suggesting that I may protect against both biotic and abiotic stress. Additionally, I is functionally involved in plant nutrition, as proteomic analysis of I-treated *Arabidopsis thaliana* plants revealed that iodinated proteins identified in the shoots were mainly associated with the chloroplast and were functionally involved in photosynthetic processes, whereas those in the roots mostly belonged and/or were related to the activities of various peroxidases.

Finally, Hu et al. highlighted and proposed *cobalt (Co)* as an essential micronutrient for plants, as it is essential for many lower plants, such as marine algal species, as well as for higher plants of the *Fabaceae* or *Leguminosae* family. Co is a component of several plant metabolic enzymes and proteins, with possible roles in key metabolisms, such as plant nitrogen fixation.

## Micronutrients: Concluding Remarks and Future Perspective

The present Research Topic provides important updates on the essential role mineral micronutrients play in plant growth and development and highlights the complex interplay of factors contributing to determine plant micronutrients availability, uptake, transport, and metabolism. Required in a narrow range of concentrations, micronutrients are a common cause of stress for plants when in deficiency or in excess. This Research Topic contributes to define micronutrients fertilization and management strategies that are critical to ensure optimal plant growth and achieve high crop yield and quality standards. Moreover, considering plants are an important dietary source of micronutrients essential for human health, this Research Topic also emphasizes the pressing need to address micronutrient deficiencies affecting a large portion of the global human population. As such, it contributes to advance the development of sustainable strategies for the biofortification of food crops. Among those, agronomic biofortification proposed as an effective strategy for enhancing the micronutrient profile of target crops, emerges in this Research Topic as a new area of research that advances our understanding of micronutrients metabolism in plants while contributing to address nutrition security issues. Finally, besides providing an update of the state of the art of micronutrient research, this Research Topic offers a perspective on future research needs and priorities. Emerging areas of research related to micronutrients include investigating (i) micronutrient roles and function in plant metabolism and their uptake and transport within the plant as a function of different genetic and environmental factors; (ii) novel fertilizer management strategies to address plant micronutrient deficiency or toxicity stress, (iii) the use of plant grafting and epigenetic technology to address micronutrients deficiency and/or toxicity stress; (iv) sustainable strategies for the development of functional food through agronomic biofortification techniques, including the use of biofertilizers, biostimulants, supplemental artificial lighting, and micronutrient nanoparticles.

## Author Contributions

All authors listed have made a substantial, direct, and intellectual contribution to the work and approved it for publication.

## Conflict of Interest

The authors declare that the research was conducted in the absence of any commercial or financial relationships that could be construed as a potential conflict of interest.

## Publisher's Note

All claims expressed in this article are solely those of the authors and do not necessarily represent those of their affiliated organizations, or those of the publisher, the editors and the reviewers. Any product that may be evaluated in this article, or claim that may be made by its manufacturer, is not guaranteed or endorsed by the publisher.
